# Ecogenomics-Based Mass Balance Model Reveals the Effects of Fermentation Conditions on Microbial Activity

**DOI:** 10.3389/fmicb.2020.595036

**Published:** 2020-12-02

**Authors:** Jinha Kim, Ran Mei, Fernanda P. Wilson, Heyang Yuan, Benjamin T. W. Bocher, Wen-Tso Liu

**Affiliations:** ^1^Department of Civil and Environmental Engineering, University of Illinois, Urbana-Champaign, Urbana, IL, United States; ^2^British Petroleum America, Petrochemicals Technology, Naperville, IL, United States

**Keywords:** ecogenomics, mass balance, microbial activity, wasted activated sludge, fermentation

## Abstract

Fermentation of waste activated sludge (WAS) is an alternative approach to reduce solid wastes while providing valuable soluble products, such as volatile fatty acids and alcohols. This study systematically identified optimal fermentation conditions and key microbial populations by conducting two sets of experiments under different combinations of biochemical and physical parameters. Based on fermentation product concentrations, methane production, and solid removal, fermentation performance was enhanced under the combined treatments of inoculum heat shock (>60°C), pH 5, 55°C, and short solid retention time (<10 days). An ecogenomics-based mass balance (EGMB) approach was used to determine the net growth rates of individual microbial populations, and classified them into four microbial groups: known syntrophs, known methanogens, fermenters, and WAS-associated populations. Their growth rates were observed to be affected by the treatment conditions. The growth rates of syntrophs and fermenters, such as *Syntrophomonas* and *Parabacteroides* increased with a decrease in SRT. In contrast, treatment conditions, such as inoculum heat shock and high incubation temperature inhibited the growth of WAS-associated populations, such as *Terrimonas* and *Bryobacter.* There were also populations insensitive to the treatment conditions, such as those related to *Microbacter* and *Rikenellaceae*. Overall, the EGMB approach clearly revealed the ecological roles of important microbial guilds in the WAS fermentation system, and guided the selection of optimal conditions for WAS fermentation in future pilot-scale operation.

## Introduction

Microorganisms play a variety of roles in the biogeochemical cycles in natural and engineered ecosystems because of the diverse activities they carry out (Madigan et al., 2018). However, due to an overreliance on DNA-based methods, such as 16S rRNA gene sequencing, many ecological studies only focus on the abundance of microorganisms, ignoring the discrepancy between presence and activity. Considering a phylotype that is detected by DNA-based methods, distinct scenarios of activity are possible. For example, it can be the DNA from a cell that is dead for up to 3 weeks (Nocker et al., 2006). It can be also from a cell that is still alive but enters dormancy, which is a non-growing inactive stage (Kamagata, 2015). Another scenario is that the population represented by the DNA can carry out very weak activity while it is in the decaying process. Finally, only a subset of the detected populations are actively playing ecological functions (Mei et al., 2016a). Such activity profiles can be especially complex in an ecosystem where strong microbial immigration is present (Mei and Liu, 2019). For example, an anaerobic sludge fermenter receives massive influx of microorganisms from distinct biological processes, such as activated sludge. While those exogenous aerobic microorganisms can still be detected as part of the fermenter microbial community (Kim et al., 2006; Lee et al., 2011; Chen et al., 2017), some of them are inactive or even dead, which would obscure the identification of the real fermentation contributors. Furthermore, engineered ecosystems, such as a sludge fermenter are always operated under varying conditions. Microbial activities are sensitive to the fluctuation of conditions, which has not been well-characterized.

While profiling microbial activity is of great importance, currently available tools are not effective enough to tackle this issue. DNA-based analyses, including 16S ribosomal RNA gene and metagenomic sequencing, only provide evidences of microorganisms that are detectable but not necessarily active. Other meta-omics approaches (e.g., metatranscriptomics, metaproteomics, and metabolomics) could shed lights on metabolic functions, but the information relies on availability of high-quality reference genomes, which are still scarce. Target-specific methods, such as fluorescence *in situ* hybridization (Sekiguchi et al., 1999), microautoradiography (Ito et al., 2011), stable isotope probing (Hatamoto et al., 2008), and nano secondary ion mass spectrometry (Li et al., 2008) require labeling of specific substrates or populations, thus cannot reveal the activities of the entire community. Overall, it remains challenging to obtain the activity profile of all the microbial populations in a complex ecosystem using these ecological tools.

An ecogenomics-based mass balance (EGMB) model has been developed recently (Saunders et al., 2015) and can be an effective approach to profile microbial activity in a complex community (Mei and Liu, 2019). It calculates the net growth rates of individual community members by taking their abundance in the influent and effluent of the system into account. Additionally, biomass concentration and system operating characters, such as sludge retention time are also considered in the calculation. As a result, populations with positive growth rates can be identified to represent active role players in the environment, whereas populations with negative growth rates likely make minimal ecological contributions. While this model has been applied in a variety of engineered ecosystems (Saunders et al., 2015; Mei et al., 2016a, 2019; Ali et al., 2019; Cheng et al., 2019; Pinel et al., 2020), the effects of environmental parameters on active microbial populations have not been examined. Sludge fermentation system harbors microbial populations with diverse activities and can be operated under varying conditions. A number of studies have investigated the relationship between the microbial community structure in a fermenter with fermentation performance, especially VFA production. For example, Chen et al. (2017) analyzed microbial community during WAS fermentation and found that the relative abundance of *Clostridia* was highly related to acidification and VFA production. Lee et al. (2011) investigated the shift in bacterial populations under varying SRTs and observed declining abundance of *Chloroflexi* and *Syntrophomonas*, but increasing abundance of *Bacteroidetes*. Liang et al. (2019) concluded that *Coprothermobacter*, *Fervidobacterium*, *Caldisericum*, and *Tepidiphilus* were the dominant bacterial genera that have the potential to generate VFAs from WAS fermentation based on their dominance in a fermenter. However, most studies only analyzed relative abundance data, whereas the complex *in situ* activities have not been considered. Therefore, it is necessary to apply the EGMB method to WAS fermentation system to differentiate populations that contribute to fermentation from those that are fermented and to examine the effects of operating conditions on microbial activity.

In this study, batch experiments of waste activated sludge (WAS) fermentation were conducted under different conditions [e.g., inoculum heat shock, pH, incubation temperature, and solids retention time (SRT)]. Fermentation performance was monitored based on volatile fatty acids (VFAs) concentration, CH_4_ production, and solid removal. After comparing the effects of single factors against control, the EGMB method was used to profile the microbial activities of different functional groups, i.e., fermentative bacteria, syntrophic bacteria, methanogenic archaea, and WAS associated populations that are fermented. The effects of fermentation conditions on microbial activities were demonstrated. In the end, the association between fermentation conditions, fermentation performance, and fermentation microbial populations was established using redundancy analysis.

## Materials and Methods

### Experiment Setup

The fermentation experiments were conducted in 250 mL bottles containing 135 mL basal medium without carbon sources (Narihiro et al., 2015). Fifteen milliliter anaerobic digester sludge taken from a local municipal wastewater treatment plant (Urbana, IL, United States) was added as the inoculum. The VSS of the digester sludge was 14.2 g/L and the pH was 7.1. The headspace of the bottle was flushed with N_2_/CO_2_ (80:20 v/v) and pressurized to 0.2 MPa before sealing. WAS was obtained from the clarifier underflow of a full-scale plant treating wastewater containing purified terephthalate acids and was shipped to the laboratory on ice overnight. The VSS of the WAS was 6.2 g/L and the pH was 6.9. WAS was added to individual bottles as the feed sludge using a syringe. The amount and interval of addition were determined based on desired SRT.

Two sets of experiments with varying operating parameters were conducted (Supplementary Table S1). Experiment I tested the effects of three biochemical parameters: inoculum heat shock, pH, and incubation temperature. The control was conducted with untreated inoculum, at neutral pH, and under an incubation temperature of 35°C. The inoculum heat shock was performed to inhibit methanogenesis and promote VFA production from fermentation (Steinbusch et al., 2009). It was achieved by treating the anaerobic digester sludge in water bath at 60, 80, or 100°C for 30 min prior to inoculation, which was determined based on our previous work (Mei et al., 2016b). The pH condition was set up by adjusting the initial pH to 5 or 9, which was shown to affect fermentation performance (Chen et al., 2007). Aliquots of hydrochloric acid (HCl) or sodium hydroxide (NaOH) solutions were injected when necessary during the incubation to maintain the pH. An incubator was used to maintain the incubation temperature at 55°C to provide a thermophilic condition than could accelerate fermentation rate (Jiang et al., 2013). The experimental design was further discussed with our industrial collaborators who provided comments on the practical feasibility of those conditions. All control and treatments were operated in duplicates and in a semi-continuous mode. The SRT of each treatment was manipulated by controlling the volume that was discarded from the reactor as well as the frequency of discarding. By completely mixing the reactor, we make sure that fraction of volume that was discarded equals to the fraction of solids that was discarded. Every 2 days, 3 mL of well-mixed liquid in each bottle was discarded and replaced with 3 mL fresh WAS, leading to an SRT of 100 days (3 mL/2 day/150 mL). In addition to the inoculum (day 0), biomass samples were collected on day 26, 28, 30, 32, 36, and 40 for microbial community analysis.

Experiment II determined the effects of SRT with optimal inoculum heat-shock (80°C), and pH at 5 selected from experiment I. The incubation temperature was set at 39°C because the WAS-generating plant can potentially operate a full-scale fermenter at 39°C in the future. The SRT ranged from 2.3 to 37.5 days by varying the feeding/discarding intervals (i.e., 2, 1, and 0.5 day) and volumes (i.e., 8, 12, and 32 mL). All treatments were operated in duplicates. Biomass samples were collected from the inoculum and on days 6, 14, 22, and 30 for microbial community analysis.

### Analytical Methods

Methane concentration in the bottle headspace was measured with a gas chromatography equipped with a packed column (Molesieve 13 × 80/100 2 m 1/8″, Resteck) and a thermal conductivity detector. Mixed liquid extracted from the bottles was centrifuge and filtered using 0.22 μm syringe filters. Acetic, propionic, butyric, and isobutyric acid (i.e., VFAs) concentrations in the filtrate were measured using a high-performance liquid chromatography equipped with an ion-exchange column (Hi-Plex H 300 × 7.7 mm, Agilent) and a UV detector. Ethanol, butanol, propanol, and isopropanol (i.e., alcohols) concentrations were measured using a gas chromatography equipped with a capillary column (HP-INNOWax 30 m × 0.32 mm × 0.25 μm, Agilent) and a flame ionization detector. Total and volatile suspended solids (TSS and VSS) were measured according to the standard protocol (Federation and Association, 2005). Solid removal efficiency was calculated based on $\frac{{\text{VSS}}_{\text{in}}-{\text{VSS}}_{\text{out}}}{{\text{VSS}}_{\text{in}}}$ (Switzenbaum et al., 2003).

### 16S rRNA Gene Sequencing and Sequence Analysis

Collected biomasses were kept in a −80°C freezer until 16S rRNA gene amplicon sequencing was performed as described previously (Mei et al., 2018). Briefly, genomic DNA was extracted using the FastDNA SPIN Kit for Soil (MP Biomedicals). 16S rRNA gene was amplified using the Bacteria/Archaea universal primer sets Univ515F/Univ909R. Purified and mixed PCR products were submitted to the Roy J. Carver Biotechnology Center at UIUC and sequenced on the Illumina MiSeq Bulk 2 × 300 nt paired-end system. Sequences were analyzed using QIIME 2 version 2018.11 (Bolyen et al., 2019). Initially, quality filtering, denoising, paired-end sequence merging and chimera removal were conducted using the dada2 plug-in which generated 100% sequence similar operational taxonomic units (OTUs) (Callahan et al., 2016). Dominant OTUs were selected as those with a relative abundance >2% in any sample and >0.1% in total populations, respectively, in experiment I and II sequence pools. Taxonomy was assigned using the SILVA132 database. Redundancy analysis (RDA) was performed using the R software version 3.5.1 (Ihaka and Gentleman, 1996) with the vegan 2.5-3 package (Oksanen et al., 2013). Student’s *t*-test, Pearson’s and Spearman’s correlation tests were conducted using R. The raw sequences obtained from this study were deposited in NCBI under the accession number PRJNA580085.

### The EGMB Calculation

Net growth rate of individual OTUs was calculated as described previously (Mei et al., 2019). Briefly, the cell count change of a given microbial population × within the bottle during a period of time Δt was contributed to by the cell count in fed biomass, discarded biomass, and net growth within the bottle. The following variables were used for the mass balance equation. *N*_*x*_ [−] is the cell count of × in the bottle; *n*_*x,d*_ [t^−1^] is the rate of × being fed into the bottle; *n*_*x,f*_ [t^–1^] is the rate of × being discarded from the bottle; the net growth of × in the bottle is expressed with a first-order reaction and a net growth rate μ_*x*_. As a result, the mass balance could be expressed as $\frac{\text{Δ}\,N_{x}}{\Delta \,t}=n_{x,f}-n_{x,d}+\mu_{x}\,N_{x}$. Assuming the community reached steady state at the end of incubation, there was no net change in cell count of × and $\frac{\text{Δ}\,N_{x}}{\Delta \,t}=0$. Therefore, μ_*x*_ can be expressed as $\mu_{x}=\frac{n_{x,d}-n_{x,f}}{N_{x}}=\frac{Q_{d}\,C_{d}\,p_{x,d}-Q_{f}\,C_{f}\,p_{x,f}}{V_{c}\,C_{c}\,p_{x,c}}=\frac{1}{S\,R\,T}-\frac{Q_{f}\,C_{f}\,p_{x,f}}{V_{c}\,C_{c}\,p_{x,c}}$, where Q is the rate of feeding/discarding biomass, C is the volatile suspended solids concentration used to approximate total cell count, and p is the relative abundance of microorganism × determined by 16S rRNA gene sequencing. Samples of the last day of both experiment I and experiment II were used for this calculation.

## Results

### Fermentation Performance Under Different Operating Parameters

**FIGURE 1:**
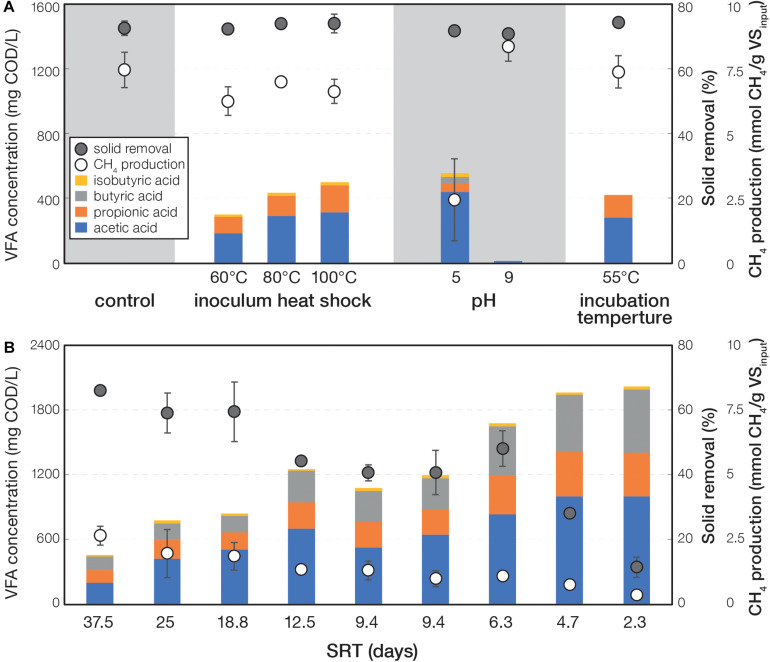
VFA concentration, CH_4_ production, and solid removal of **(A)** experiment I at day 24 and **(B)** experiment II at day 18.

The parameters tested in this study (i.e., inoculum heat shock, pH, and incubation temperature in experiment I and SRT in experiment II) led to distinct fermentation performance compared to control. In experiment I, the control and treatment under pH 9 did not accumulate VFA throughout the incubation (Supplementary Figure S1). The remaining treatments all showed varying degrees of VFA accumulation, which decreased toward the end of the incubation in concordance with increasing methane accumulation. Such features suggested a typical performance of batch reactors. At day 24 of experiment I when most cultures reached maximum VFA accumulation, the treatments under inoculum heat shock, pH 5, and 55°C incubation temperature had higher VFA accumulation compared to control (Figure 1A and Supplementary Table S2). Acetic acid was the major VFA component (184–438 mg COD/L), followed by propionic acid (55–167 mg COD/L), butyric acid (only in pH 5 at 40 mg COD/L), and isobutyric acid (17–23 mg COD/L). Alcohols were not detected in all treatments. Methane production was observed to range from 6.3 to 8.5 mmol CH_4_/g VS_input_ for all the treatments, except for the condition at pH 5 (2.5 mmol CH_4_/g VS_input_). All treatments had solid removal efficiencies ranging from 71.3 to 77.2%. In experiment II that determined the effects of SRT (2.5–37.5 days), most treatments exhibited the highest VFA concentrations at day 18, which decreased toward the end of incubation (Supplementary Figure S2). At day 18, shorter SRT were observed to have more VFA accumulation, less CH_4_ production, and less solid removal (Figure 1B and Supplementary Table S2). The shortest SRT (i.e., 2.3 days) produced the maximum VFA concentration of 2,002 mg COD/L, and 37.5 days SRT yielded the maximum CH_4_ production (0.16 mmol CH_4_/g VS_input_) and solid removal (65%).

### Conventional Analysis of Microbial Community Structures

Microbial community was analyzed using conventional diversity analyses. For experiment I, biochemical parameters had clear effects on the community beta-diversity (Supplementary Figure S3A). The control and the treatment at pH 9 clustered together and were adjacent to the inocula. The treatments with inoculum heat shock, low pH, and 55°C incubation temperature were distant from the control. Based on the relative abundance of individual OTUs, communities in the inoculum, control, and pH 9 treatment contained higher abundance of syntrophs and methanogens than other treatments (Supplementary Figure S4). Clearly, treatments with inoculum heat shock, low pH, and 55°C incubation temperature tended to suppress methanogenic process compared to control. The abundance of other OTUs was also affected by the treatments. For example, inoculum heat shock decreased the abundance of OTUs related to *Bacteroidales* (OTU3, *p* = 10^–5^), *Rikenellaceae* (OTU2, *p* = 3.6 × 10^–5^), and *Ca.* Cloacimonetes (OTU6, *p* = 10^–7^, and 125, *p* = 0.0008), but increased the abundance of other OTUs (OTU285, *p* = 9 × 10^–5^, 147, *p* = 0.008, and 182, *p* = 10^–5^) in comparison to control. The treatment of pH 5 increased the abundance of OTUs related to *Microbacter* (OTU908, *p* = 0.004) and *Parabacteroides* (OTU909, *p* = 0.005), and 55°C incubation temperature increased the abundance of OTUs related to uncultured *Bacteroidales* (OTU901, *p* = 10^–5^) and *Fervidobacterium* (OTU928, *p* = 0.006, 929, *p* = 0.003, 930, *p* = 10^–5^ and 931, *p* = 0.009) that belonged to *Thermotogae*.

For experiment II, SRT was not observed to have clear effects on community structures and communities with the same SRT did not cluster together tightly (Supplementary Figure S3B and Supplementary Table S3). Based on relative abundance, syntrophs and methanogens were present in low abundance (<0.5%) in all samples, suggesting they were not adapted to these SRTs (Supplementary Figure S5). The abundance of other major populations changed in a concurrent manner and was not affected by SRT. Clearly, based on differences in relative abundance, the effects of the SRT on community structure dynamics in experiment II could not be easily discerned.

### EGMB Model Revealed the Effects of Treatment Conditions on Microbial Activity

**FIGURE 2:**
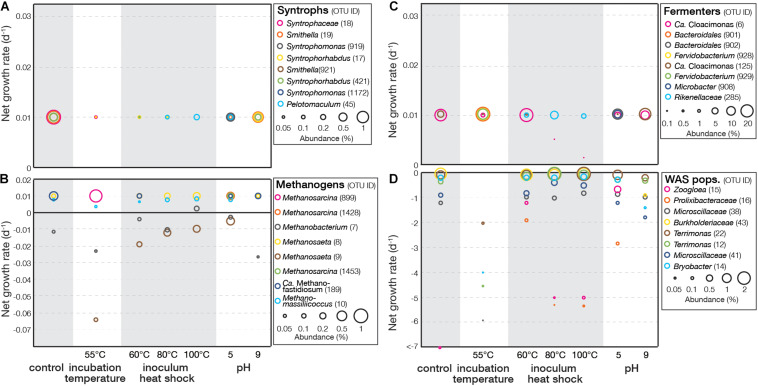
Net growth rate of representative OTUs (top eight abundant) related to syntrophs, methanogens, fermenters, and WAS populations in **(A–D)** experiment I.

For experiment I, the EGMB model was applied to identify active populations in the last day of the experiment (Supplementary Figure S6). Known syntrophs and known methanogens were identified based on their phylogenetic affiliation. For the remaining OTUs, those with negative growth rates were identified to primarily associate with WAS populations and those with positive growth rates were identified as fermenters based on their phylogenetic relation with the sequences of known fermenters. Figure 2A showed the effects of treatment conditions on the growth rates of representative syntrophs (top eight abundant OTUs). Although their abundance was generally lower than 1%, their growth rates reached 0.01 d^–1^ that was the maximum value under the SRT and were not affected by the treatment conditions. The growth rates of methanogens varied widely (Figure 2B). Some remained positive and was not affected by the treatments, including those related to *Methanosaeta* (OTU8), *Methanomassiliicoccus* (OTU10), and *Ca.* Methanofastidiosum (OTU189). Others were sensitive to the treatments, such as OTU7 related to *Methanobacterium* and OTU9 related to *Methanosaeta* that showed decreasing growth rates at high incubation temperature and inoculum heat shock. At 55°C incubation temperature, a significant decrease in growth rate or no detection was observed for six of the eight methanogens, suggesting these methanogens were inhibited by the high incubation temperature. In contrast, OTU899 related to *Methanosarcina* showed high growth rate at 55°C incubation temperature, and OTU10 related to *Methanomassiliicoccus* was only slightly affected, suggesting they were thermophilic methanogens.

Fermenters exhibited a similar pattern with syntrophs with most having a maximum growth rate of 0.01 d^–1^ (Figure 2C). The growth rates of OTU6 related to *Ca*. Cloacamonas were inhibited by inoculum heat shock, suggesting its sensitivity to such treatments. WAS populations with negative growth rates were primarily associated with aerobic microbial taxa (Figure 2D). Populations related to *Terrimonas* (OTU12 and 22), *Bryobacter* (OTU14), and *Microscillaceae* (OTU38 and 41) exhibited lower growth rates at high incubation temperature, indicating this treatment could enhance the decomposition of these populations. In contrast, a *Zoogloea*-related population (OTU15) had the lowest growth rate in control, suggesting all treatment conditions tested did not further enhance the decomposition.

**FIGURE 3:**
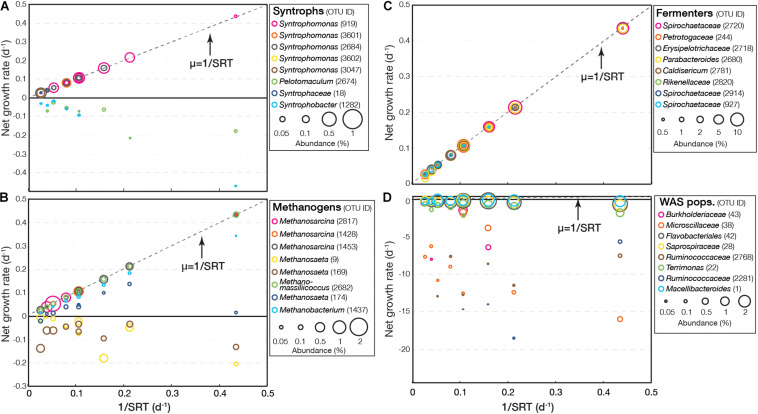
Net growth rate of representative OTUs (top eight abundant) related to syntrophs, methanogens, fermenters, and WAS-associated populations in **(A–D)** experiment II.

For experiment II, the EGMB model clearly revealed the impacts of SRT on different microbial groups (Figure 3 and Supplementary Figure S7). As shown in Figure 3A, three distinct types of growth rates were observed for syntrophs. The first type, represented by OTUs related *Syntrophomonas* (OTU919 and 2684), had positive growth rates, which increased with a decrease in SRT and aligned tightly with the maximum value of 1/SRT. The second type had positive growth rates at long SRT but was not detected under short SRT, including those related to *Syntrophomonas* (OTU3601, 3602, and 3047) and *Syntrophaceae* (OTU18). Likely, they were washed out from the system under short SRT. The third type had negative growth rates that decreased with a decrease in SRT but was still detected. This type included OTUs related to *Pelotomaculum* (OTU2674) and *Syntrophobacter* (OTU1282). The profile of methanogens were similar to that of syntrophs (Figure 3B). Most methanogens had positive growth rates and increased with a decrease in SRT, including *Methanosarcina* (OTU2871, 1423, and 1453), *Methanomassiliicoccus* (OTU2682), *Methanosaeta* (OTU174), and *Methanobacterium* (OTU1437). Two OTUs related to *Methanosaeta* (OTU9 and 169) had negative growth rates that decreased with a decrease in SRT, indicating slow growth of these populations.

Fermenters, such as *Parabacteroides* (OTU2680), *Rikenellaceae* (OTU2820), and *Spirochaetaceae* (OTU2720, 2914, and 927) constantly had positive growth rates (Figure 3C). Their growth rates increased with respect to a decrease in SRT and their growth rates aligned tightly with the maximum value under the given SRT (i.e., 1/SRT), suggesting shorter SRTs tended to promote the growth of fermenters. WAS populations consistently had negative growth rates and the growth rates of some populations decreased with a decrease in SRT, suggesting enhanced decomposition (Figure 3D). Among them, the decrease in growth rate of a *Microscillaceae*-related OTU (OTU38) and a *Ruminococcaceae*-related OTU (OTU2281) was most obvious (to < −15 d^–1^). In contrast, OTU42 related to *Flavobacteriales*, OTU28 related to *Saprospiraceae*, and OTU1 related to *Macellibacteroides* had negative growth rates but close to zero, suggesting they were more resistant in the fermentation culture.

### The Association Between Fermentation Condition, Performance, and Active Populations

**FIGURE 4:**
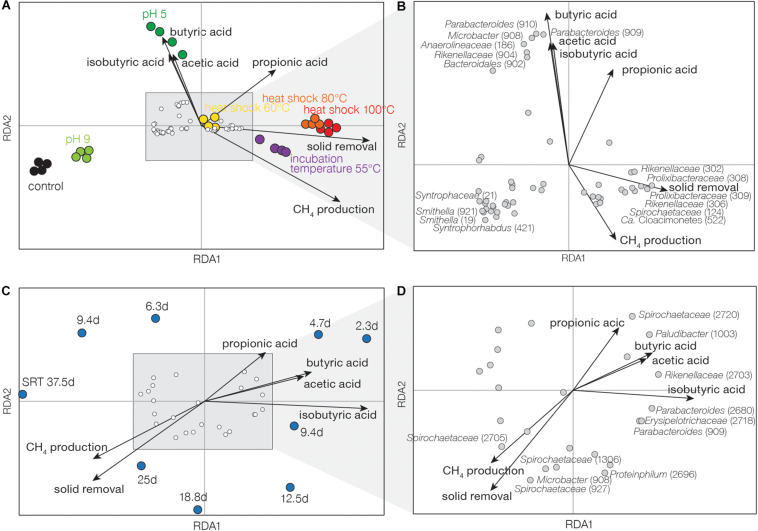
Redundancy analysis based on active and dominant OTUs of **(A,B)** experiment I and **(C,D)** experiment II. **(A,C)** are tri-plots of entire communities, OTUs, and performance data. **(B,D)** are zoomed in plots of OTUs and performance data. Length and direction of arrows indicate the strength and intensity of the performance data. OTU ID is labeled.

RDA was further performed to identify the association between the operational conditions (inoculum heat shock, pH, incubation temperature, and SRT), fermentation performance (VFA concentration, CH_4_ production, and solid removal), and the abundance of microbial populations with positive growth rates. In experiment I, VFA concentrations were highly correlated with low pH treatment (Figure 4A). Solid removal and CH_4_ production were associated with incubation temperature at 55°C and inoculum heat shock of 80 and 100°C. Six OTUs were found to be closely correlated with VFA concentrations (Figure 4B) and they were associated with *Parabacteroides* (OTU909 and 910), *Microbacter* (OTU908), *Anaerolineaceae* (OTU186), *Rikenellaceae* (OTU904), and *Bacteroidales* (OTU902). OTUs related to syntrophs were found in the opposite direction of VFA concentrations, including *Syntrophorhabdus* (OTU421), *Smithella* (OTU921 and 19), and *Syntrophaceae* (OTU21). Other OTUs were associated with solid removal, including *Rikenellaceae* (OTU302 and 306), *Prolixibacteraceae* (OTU308 and 309), *Ca.* Cloacimonetes (OTU522), and *Spirochaetaceae* (OTU124). In experiment II, high SRTs were associated with higher CH_4_ production and solid removal, and low SRT treatments were related to higher VFA concentrations (Figure 4C). Like experiment I, we observed OTUs associated with VFA concentrations (Figure 4D), including *Parabacteroides* (OTU909 and 2680), *Rikenellaceae* (OTU2703), *Paludibacter* (OTU1001), *Spirochaetaceae* (OTU2703), and *Erysipelotrichaceae* (OTU2718). Other populations, such as *Microbacter* (OTU908), *Proteiniphilum* (OTU2696), and *Spirochaetaceae* (OTU2705, 927, and 1306) were associated with CH_4_ production and solid removal.

## Discussion

Anaerobic fermentation of WAS has been intensively investigated (Ahn and Speece, 2006; Kim et al., 2006; Chen et al., 2007, 2017; Zhang et al., 2009; Lee et al., 2011; Xiong et al., 2012; Vanwonterghem et al., 2015; Yuan et al., 2015), but few study can identify active and inactive microbial populations within the system. This is caused by the fact that conventional community structure analysis by 16S rRNA gene alone can only detect the presence of a community member but not the activity. Such phenomenon was also observed in this study where simple examination of beta-diversity and relative abundance profile failed to reveal the effects of operation parameters especially SRT on microbial community dynamics. Considering fermenters and other wastewater treatment bioreactor receive intense microbial immigration from the upstream processes (Mei and Liu, 2019), microbes that are detected in the reactor do not necessarily contribute to the functions of the reactor. Therefore, it is essential to effectively differentiate active and inactive populations in such immigration-heavy systems. This task can be achieved by applying the EGMB model, which allowed us to successfully pinpoint populations that were fermented based on their negative growth rates. The results obtained in this study are also in line with a number of recent reports that microbial communities in engineered water environments contain significant abundance of inactive populations, which should be carefully analyzed (Saunders et al., 2015; Mei et al., 2016a, 2019; Ali et al., 2019; Cheng et al., 2019; Pinel et al., 2020).

Furthermore, we clearly revealed the effects of various operating parameters on the activities of known syntrophs, known methanogens, fermenters, and WAS-associated populations. Some syntrophs and methanogens were inhibited, including those related to *Syntrophaceae*, *Pelotomaculum*, and *Methanosaeta* under shorter SRTs, suggesting these slow-growing anaerobes were washed out. Other species showed increasing growth rates with a decrease in SRT (e.g., *Syntrophomonas* and *Methanomassiliicoccus*), suggesting they had growth advantage over other slow-growing population that occupied the same niche. We also observed heterogeneous growth rates within the same genus. For example, multiple OTUs related to *Methanosaeta* exhibited distinct growth rate pattern under the effect of SRT, i.e., two OTUs had decreased growth rates, whereas another one had increased growth rates along with a decrease in SRT. Populations introduced by WAS were consistently found to have negative growth rates in all conditions, suggesting they were decomposed during fermentation. They were associated with aerobic microbial taxa, such as *Terrimonas* (Zhang et al., 2012) and *Bryobacter* (Kulichevskaya et al., 2010). High incubation temperature and shorter SRT could lead to lower growth rates of most WAS populations, indicating these conditions were favorable for biomass decomposition. In contrast, the growth rate of a *Zoogloea*-related population was not significant different from that observed in the control, likely due to the reason that these populations can utilize storage polymers or extracellular polysaccharides as mechanisms to resist fermentation (Dugan et al., 2006). Among populations with positive growth rates, fermentative organisms accounted for the majority of the community. This included OTUs related to *Microbacter*, *Fervidobacterium*, and *Parabacteroides* that were known to ferment sugars (Patel et al., 1985; Tan et al., 2012; Sanchez-Andrea et al., 2014), *Rikenellaceaea* that was frequently observed in methanogenic environments (Su et al., 2014; Mei et al., 2017), and *Ca.* Cloacimonetes that were proposed with various anaerobic functions (Pelletier et al., 2008; Lykidis et al., 2010; Ju and Zhang, 2014; Nobu et al., 2015; Dyksma and Gallert, 2019). The growth rates of fermenters were further observed to increase with a decrease in SRT. In total, these results indicated that evaluating *in situ* activity could provide accurate understanding of the ecological roles of different microbial guilds than simply examining relative abundance.

Redundancy analysis between fermentation performance and microbial populations, especially those with positive growth rates, further revealed the potential contribution of key organisms in the fermentation process. For example, *Parabacteroides* and *Paludibacter* populations were found to be closely associated with the accumulation of VFAs in both experiment I and experiment II, indicating they were the key VFA producers. Such findings were further supported by the fact that isolated species of *Parabacteroides* and *Paludibacter* were known to ferment various types of carbohydrates and produce acetate, propionate and other fatty acids (Ueki et al., 2006; Wang et al., 2019). These populations were also found in other anaerobic environments as dominant community members, such as *Parabacteroides* in WAS digestion (Mei et al., 2016a) and *Paludibacter* in WAS fermentation (Yuan et al., 2015). In addition, syntrophs were found to be negatively associated with VFA accumulation in experiment I, suggesting their function of consuming VFAs were inhibited by the biochemical parameters. These results demonstrate that our approaches effectively identified key functional contributors and revealed their potential functions in a complex community.

Overall, we found that inoculum heat shock, pH 5, 55°C incubation temperature, and shorter SRT were individually favorable to maximize fermentation products accumulation, which could be used to guide the operation of larger scale industrial installations to enhance fermentation performance. Meanwhile, we are aware that further investigations are required to overcome several limitations of this study. One of our limitations was that we did not take a full factorial design or a fractional factorial design considering the large number of factors, levels, and replicates. Therefore, we could not make any conclusion about the relative importance of each factors and the interactions between them. The small reactor volume (250 mL), short operation period (30–40 days), and semi-continuous feeding mode may not fully represent the situation in a continuously operated reactor at a larger scale. Herein, the optimal conditions obtained from this study have been used as the reference for a lab-scale fermenter that receives constant sludge feeding in our follow-up research. As for the EGMB model, it could effectively differentiate active microbial populations from WAS associated biomass, and further reveal different responses of growth rate to treatment conditions. The model clearly provides higher resolution to the microbial dynamics than 16S rRNA gene sequencing alone, and can be applied to diverse engineered water systems to unravel the overlooked diversity of microbial activities. On the other hand, the EGMB model should be used with cautions considering its methodological limitations. As we have reviewed recently (Mei and Liu, 2019), 16S rRNA gene based abundance calculation suffers from copy number issues and could be potentially substituted by metagenomic sequencing. Volatile solids-based biomass quantification can be improved by using flow cytometry and microscopic enumeration. More efforts are needed in the future using the EGMB approach and other ecological methods to better understand the diversity of microbial activities in a wide variety of ecosystems.

## Data Availability Statement

The raw sequences obtained from this study were deposited in NCBI under the accession number PRJNA580085.

## Author Contributions

JK, RM, FW, BB, and W-TL designed the study. JK, RM, and BB performed the experiment. JK and RM analyzed the data and wrote the manuscript. RM, BB, and W-TL revised the manuscript. All authors contributed to the article and approved the submitted version.

## Conflict of Interest

BB was employed by the company British Petroleum. The remaining authors declare that the research was conducted in the absence of any commercial or financial relationships that could be construed as a potential conflict of interest.
