# Supplementary material for: Ecogenomics-Based Mass Balance Model Reveals the Effects of Fermentation Conditions on Microbial Activity
Source: Front Microbiol. 2020 Dec 2;11:595036. doi: 10.3389/fmicb.2020.595036 (PMC7738435; doi:10.3389/fmicb.2020.595036)
Supplement: Supplementary file 1 [file Data_Sheet_1.pdf]

Supplementary Material

1 Supplementary Figures and Tables

1.1 Supplementary Figures

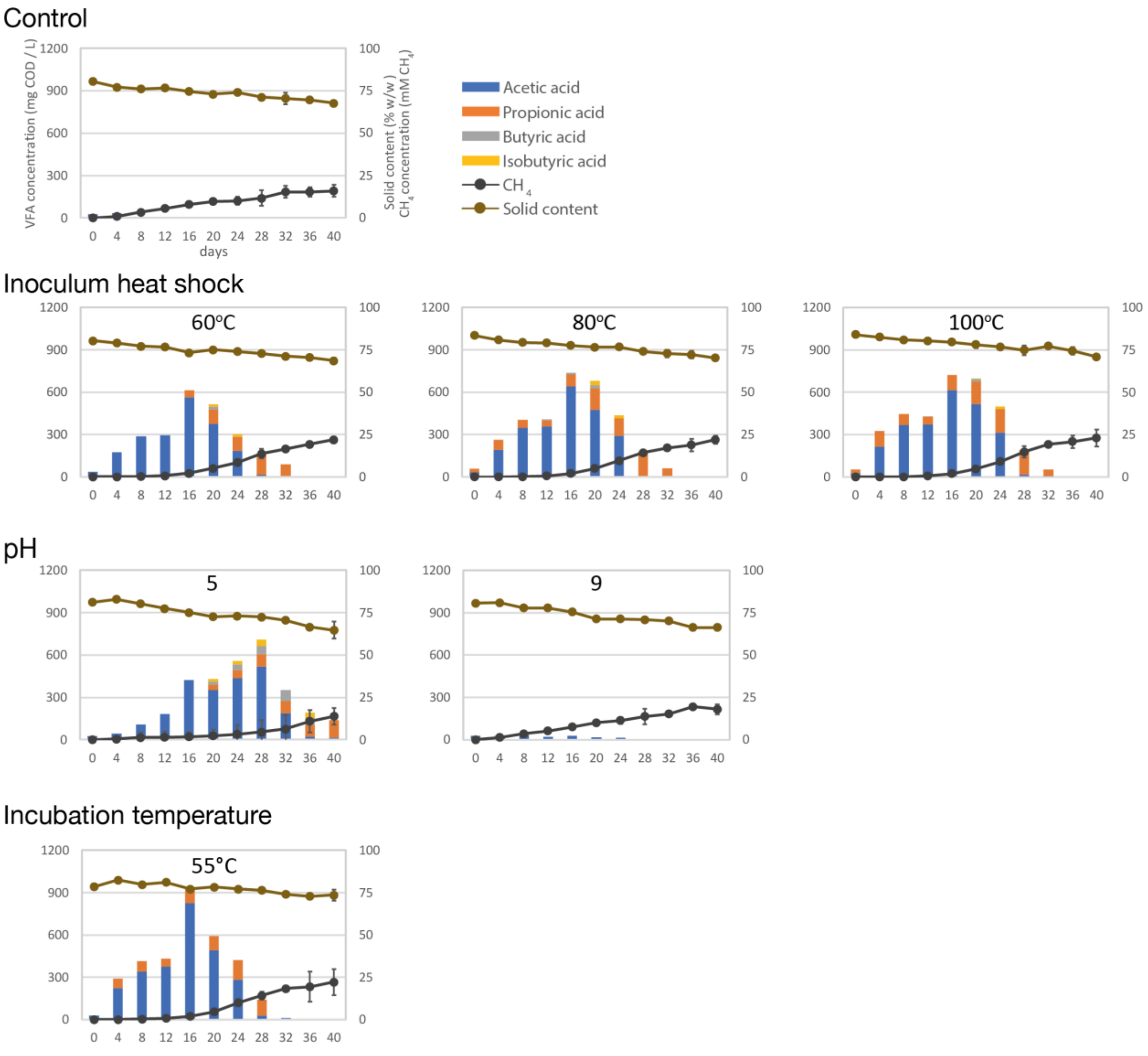

Supplementary Figure 1. VFA concentration, CH_4 concentration and solid content profile by biochemical treatment effects (e.g., inoculum heat shock, pH and incubation temperature) in experiment I.

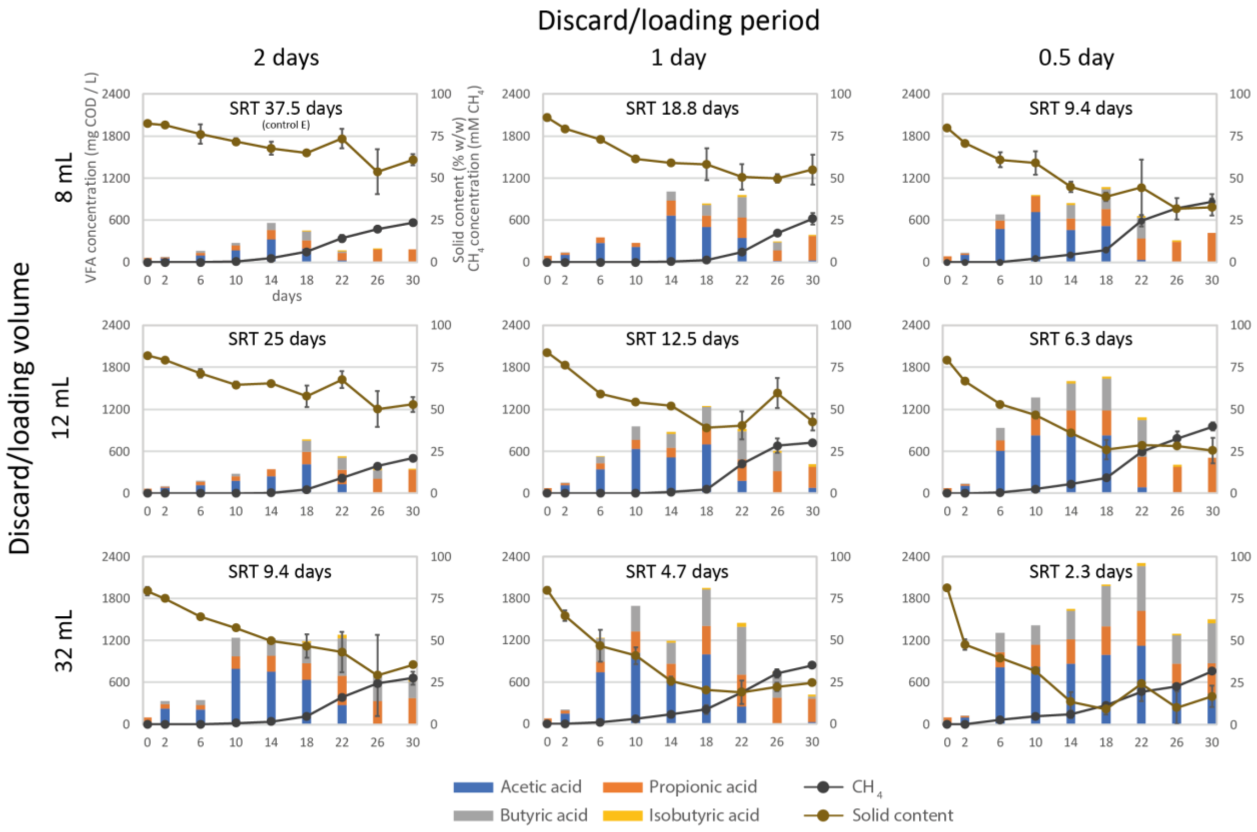

Supplementary Figure 2. VFA concentration, CH_4 concentration and solid content profile by SRT in experiment II.

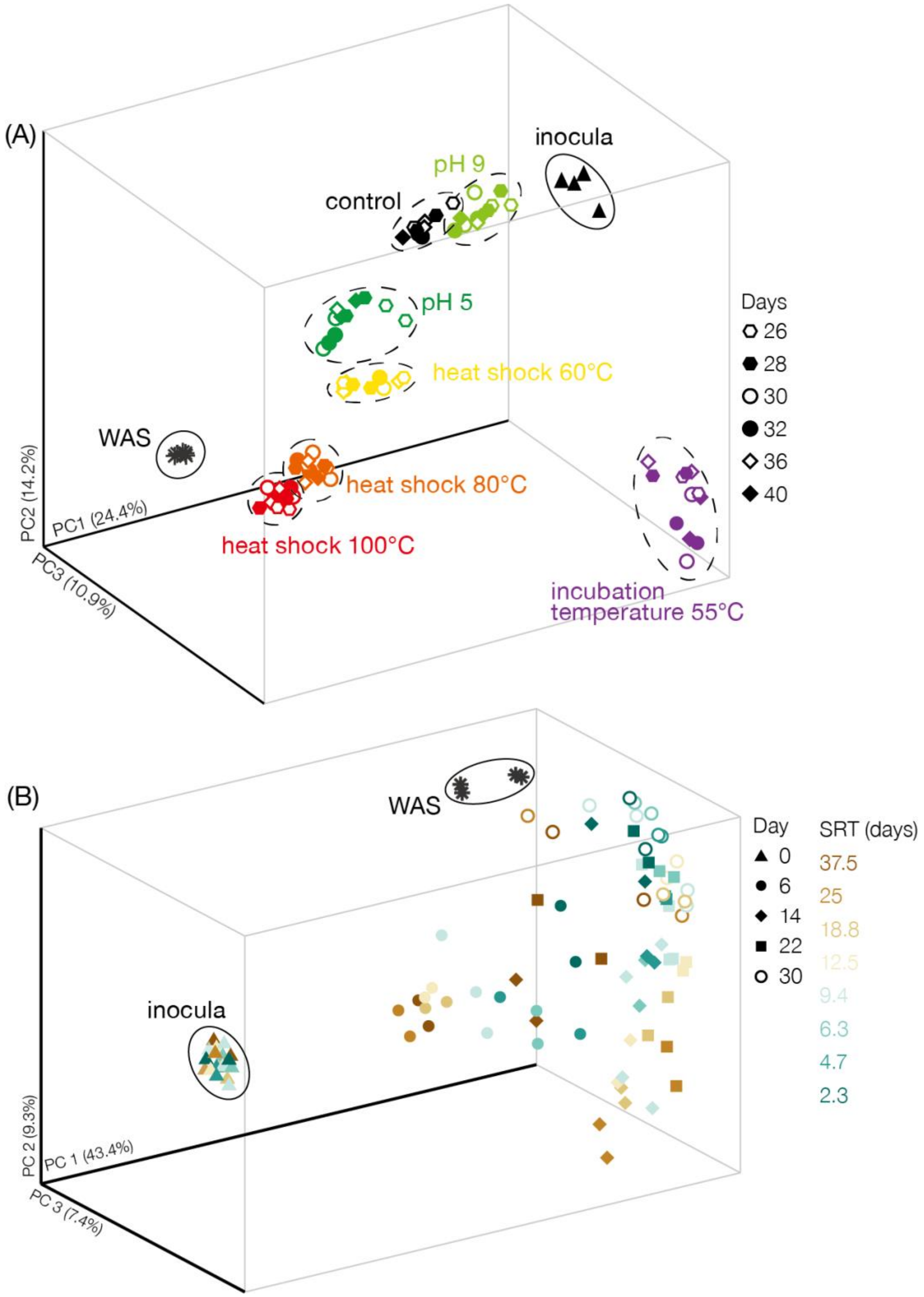

Supplementary Figure 3. Beta-diversity of (A) experiment I and (B) experiment II based on principal coordinate analysis of unweight Unifrac distance matrices.

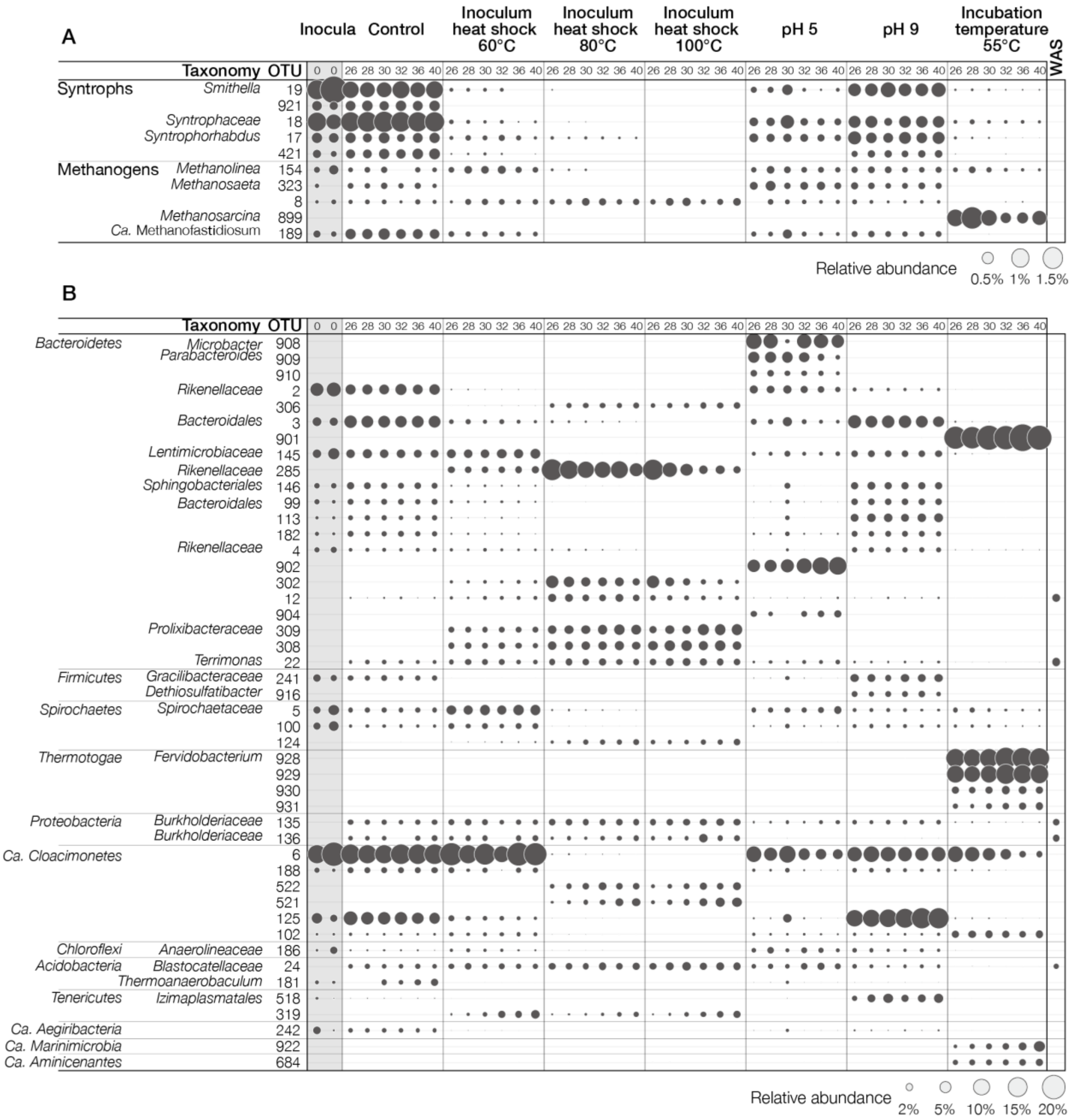

Supplementary Figure 4. Relative abundance of major OTUs in experiment I. Five syntrophs and 5 methanogens (<2% in sample, >0.1% of total population) and 46 other bacterial OTUs (>2% in sample, >0.1% of total population) are shown.

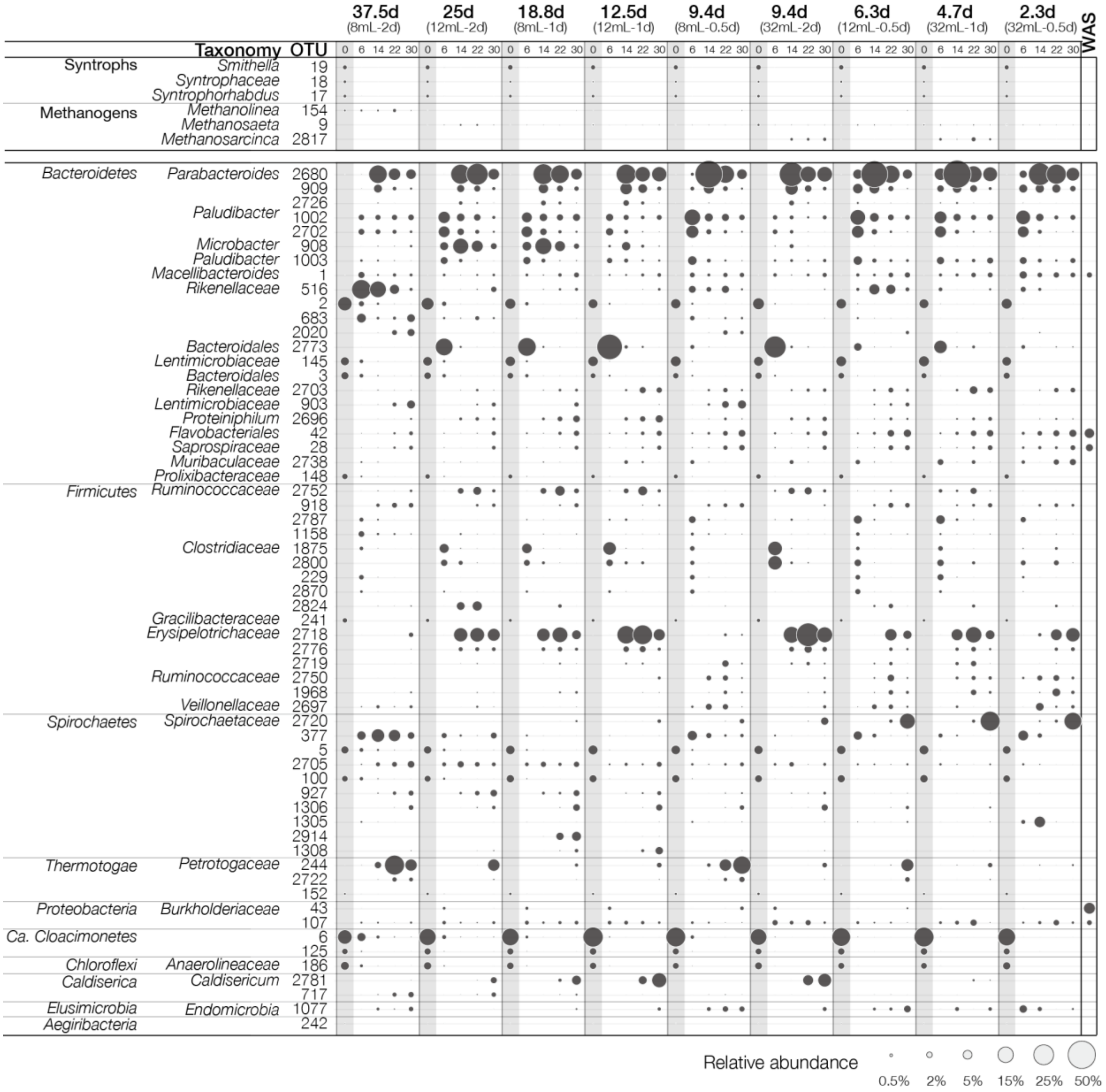

Supplementary Figure 5. Relative abundance of major OTUs in experiment II. Three syntrophs and methanogens (<2% in sample, >0.1% of total population) and 60 predominant bacterial OTUs (>2% in sample, >0.1% of total population) are shown.

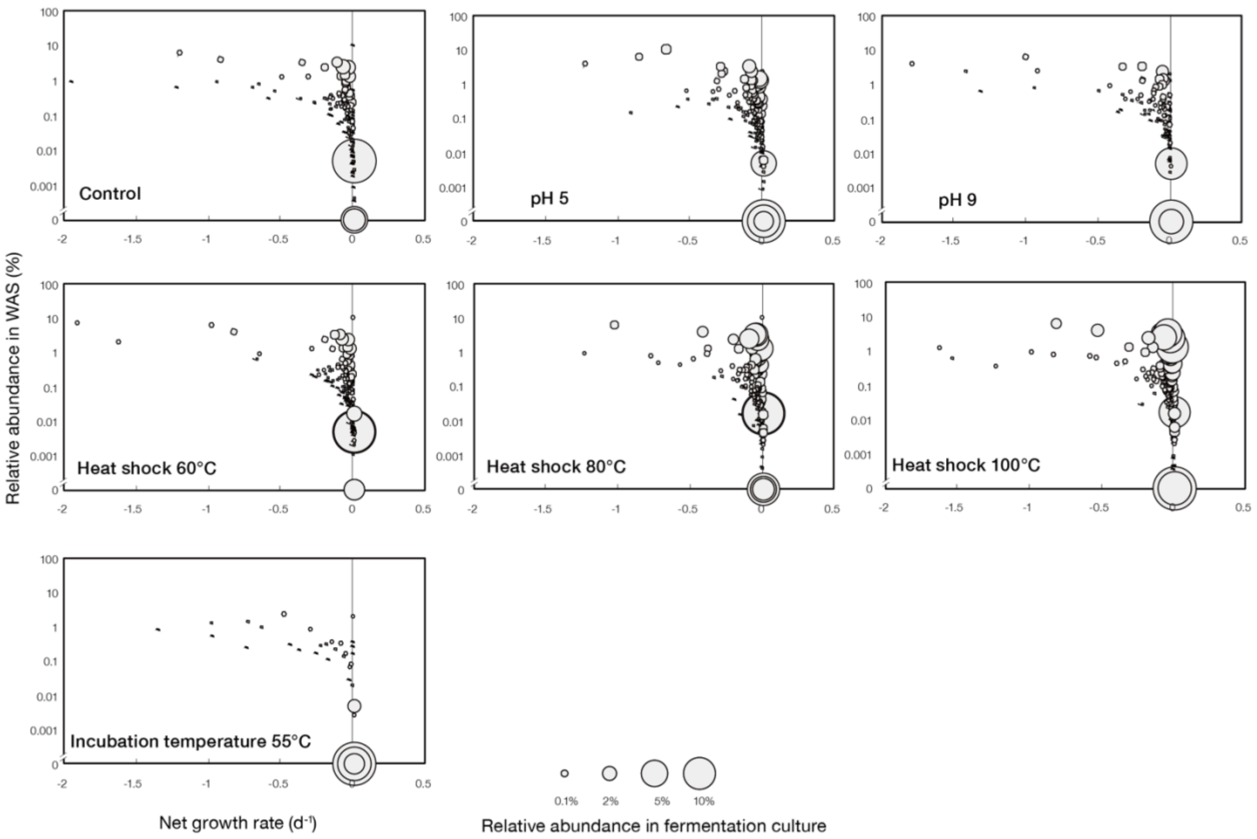

Supplementary Figure 6. Distribution of net growth rate in experiment I at day 40 under different treatments.

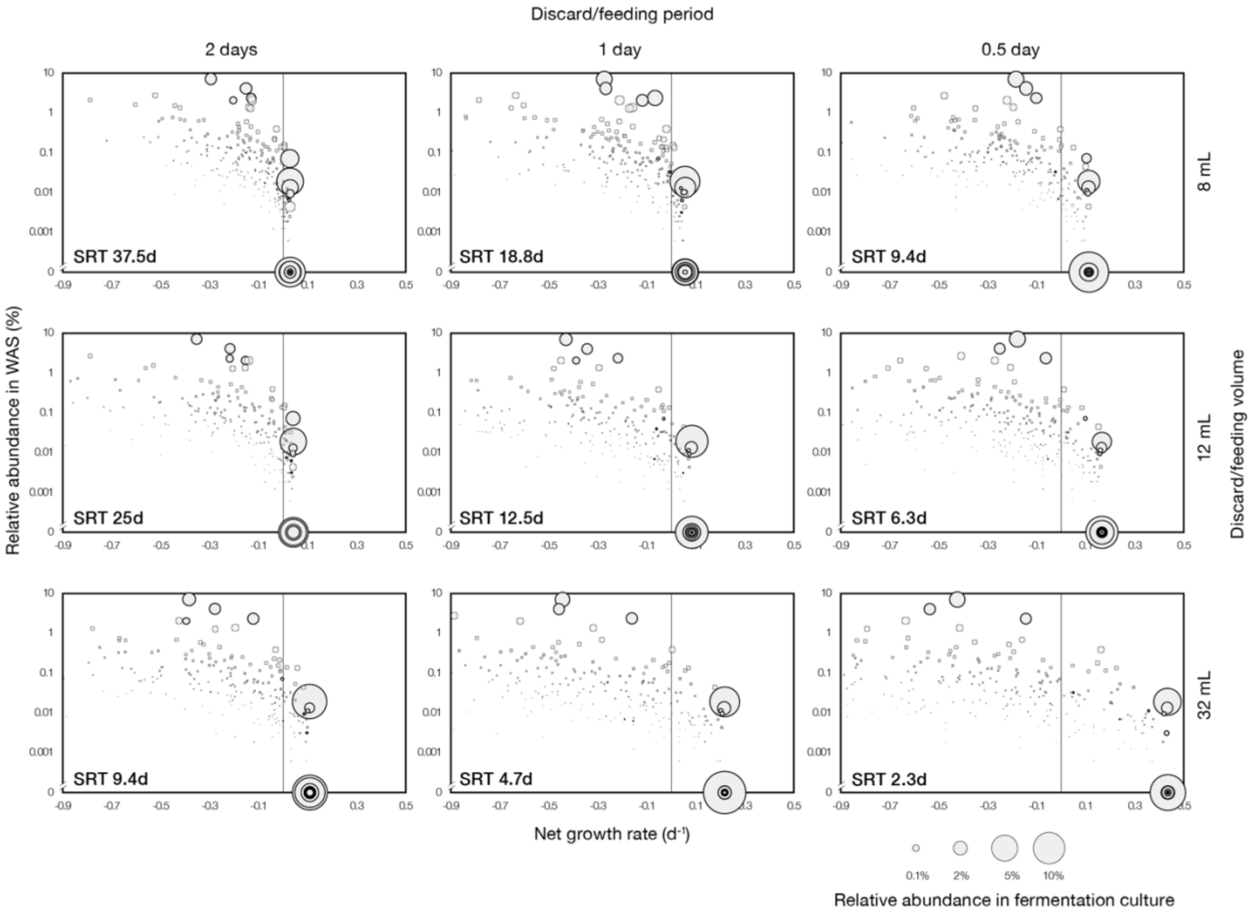

Supplementary Figure 7. Distribution of net growth rate in experiment II at day 30 under different SRTs.

1.2 Supplementary Tables (Data Sheets)

Data Sheet 1. Operational conditions tested in this study

	Treatment condition	Inoculum heatshock (°C)	pH	Incubation temperature (°C)	SRT (day)
**Experiment I**	Control	NA	7	35	100
	Inoculum heatshock 60°C	60	7	35	100
	Inoculum heatshock 80°C	80	7	35	100
	Inoculum heatshock 100°C	100	7	35	100
	pH 5	NA	5	35	100
	pH 9	NA	9	35	100
	Incubation temperature 55°C	NA	7	55	100
**Experiment II**	SRT 37.5 day	80	5	39	37.5
	SRT 25 day	80	5	39	25
	SRT 18.8 day	80	5	39	18.8
	SRT 12.5 day	80	5	39	12.5
	SRT 9.4 day (8mL-0.5d)	80	5	39	9.4
	SRT 9.4 day (32mL-2d)	80	5	39	9.4
	SRT 6.3 day	80	5	39	6.3
	SRT 4.7 day	80	5	39	4.7
	SRT 2.3 day	80	5	39	2.3

Data Sheet 2. P-values of t test for fermentation performance

	**Treatment condition**	VFA accumulation	Methane accumulation	Solid removal
**Experiment I**	Control	-	-	-
	Inoculum heatshock 60°C	0.002	0.194	0.026
	Inoculum heatshock 80°C	0.014	0.461	0.059
	Inoculum heatshock 100°C	0.001	0.293	0.027
	pH 5	0.016	0.054	0.803
	pH 9	0.134	0.288	0.137
	Incubation temperature 55°C	0.023	0.918	0.036
**Experiment II**	Control (SRT 37.5 day)	-	-	-
	SRT 25 day	0.114	0.082	0.965
	SRT 18.8 day	0.016	0.022	0.605
	SRT 12.5 day	0.091	0.048	0.070
	SRT 9.4 day (8mL-0.5d)	0.012	0.399	0.162
	SRT 9.4 day (32mL-2d)	0.008	0.221	0.261
	SRT 6.3 day	0.019	0.055	0.241
	SRT 4.7 day	0.018	0.362	0.055
	SRT 2.3 day	0.008	0.045	0.013

Data Sheet 3. P-values of PERMANOVA test for community similarity in experiment II on day 30

	SRT 37.5 day	SRT 25 day	SRT 18.8 day	SRT 12.5 day	SRT 9.4 day (8mL-0.5d)	SRT 9.4 day (32mL-2d)	SRT 6.3 day	SRT 4.7 day	SRT 2.3 day
SRT 37.5 day	-								
SRT 25 day	0.64	-							
SRT 18.8 day	0.321	0.665	-						
SRT 12.5 day	0.312	0.672	0.675	-					
SRT 9.4 day (8mL-0.5d)	0.339	0.336	0.337	0.344	-				
SRT 9.4 day (32mL-2d)	0.332	0.362	0.369	0.317	0.356	-			
SRT 6.3 day	0.346	0.37	0.343	0.326	0.315	0.341	-		
SRT 4.7 day	0.302	0.338	0.318	0.342	0.324	0.339	0.349	-	
SRT 2.3 day	0.36	0.337	0.336	0.338	0.322	0.343	0.34	0.35	-

1.3 Supplementary method: the ecogenomics-based mass balance calculation

The batch is operated semi-continuously. Thus, only during the time when the samples are discarded from the batches are subjected to the mass balance calculation. The net change in cell count of microorganisms x could be described as the following equation representing a semi-continuous mass balance with assumption of the net growth being explained as a first order process.

$$\left.\frac{\Delta N_{x,batch}}{\Delta t}\right|_{t+\Delta t} = \mu_{x,batch} N_{x,batch,t+\Delta t} + n_{x,in,t} - n_{x,out,t+\Delta t} \text{ (S1)}$$

where,

$N_{x,batch}$: absolute cell count of microorganism x in batch [-]

μ_x : net specific growth rate of microorganism x [t^{-1}]

$n_{x,in}$: cell count of microorganism x in waste activated sludge loaded into batch per day [t^{-1}]

$n_{x,out}$: cell count of microorganism x in discarded sample from batch per day [t^{-1}]

Δt : sludge discarding and loading period [t]

As the batch reaches a semi-steady state (presumably last day of experiment), no net change in cell count of x is assumed during at discarding moments. Hence, $\left.\frac{\Delta N_{x,batch}}{\Delta t}\right|_{t+\Delta t} = 0$ would be assumed for every discarding moments which allows the rearrangement of the mass balance equation (S1)

$$\mu_{x,batch} = \frac{n_{x,out,t+\Delta t} - n_{x,in,t}}{N_{x,batch,t+\Delta t}} \text{ (S2)}$$

Cell count of x in discarded sludge, WAS and the working volume of batch could be calculated with relative abundance of x (p_x) obtained from the OTU table multiplied to the total cell number which expands the equation (S2)

$$\mu_{x,batch} = \frac{p_{x,out,t+\Delta t} n_{out,t+\Delta t} - p_{x,in,t} n_{in,t}}{p_{x,out,t+\Delta t} N_{batch,t+\Delta t}} \text{ (S3)}$$

$n_{out,t+\Delta t}$ and $n_{in,t}$ are total cell counts leaving and introduced into the system per day. $n_{out,t+\Delta t}$ could be calculated by multiplying the cell concentration (L^{-1}) in the discarded sample (C_{out}) with the daily volume of discarded sample $Q_{out,t+\Delta t}$. $n_{in,t}$ is calculated similarly by multiplying the cell concentration in the WAS with the daily volume of WAS introduced $Q_{in,t}$. $Q_{in,t} = Q_{out,t+\Delta t} = Q$ since working volume should maintain the same. $N_{batch,t+\Delta t}$ is the total cell count in the batch at the moment of discarding and could be calculated by multiplying the working volume of the batch V_{batch} and cell concentration in the discarding sample $C_{batch,t+\Delta t}$. $p_{x,out,t+\Delta t} = p_{x,batch,t+\Delta t}$ and $C_{out,t+\Delta t} = C_{batch,t+\Delta t}$ as semi-steady state considers same sample concentrations of the discarding sample with what was present in the batch. This again expands and simplifies the equation (S3)

$$\mu_{x,batch} = \frac{p_{x,out,t+\Delta t}Q_{out,t+\Delta t}C_{out,t+\Delta t} - p_{x,in,t}Q_{in,t}C_{in,t}}{p_{x,batch,t+\Delta t}V_{batch}C_{batch,t+\Delta t}} = \frac{Q}{V_{batch}}\left(\frac{p_{x,out,t+\Delta t}C_{out,t+\Delta t} - p_{x,in,t}C_{in,t}}{p_{x,batch,t+\Delta t}C_{batch,t+\Delta t}}\right) = \frac{Q}{V_{batch}}\left(1 - \frac{p_{x,in,t}C_{in,t}}{p_{x,out,t+\Delta t}C_{out,t+\Delta t}}\right) \text{ (S4)}$$

From this final equation, concentration of volatile solids VS (g VS / L) of the sludge is used as a proxy for cell concentration ($C_{in,t}$, $C_{out,t+\Delta t}$) (e.g. $C_{in,t} = VS_{in,t}$, $C_{out,t+\Delta t} = VS_{out,t+\Delta t}$). This was previously verified with the use of qPCR to validate the usage of the VS value to estimate cell count for mass balance (Mei et al., 2016). VS is calculated by multiplying the total solid concentration (g TS / L) with the volatile solid fraction (% VS). For further simplification, $\frac{Q}{V_{batch}} = \frac{1}{SRT}$ since HRT=SRT for the fed batch system. Thus, a final equation could be expressed as the following for the semi-continuously fed batch system.

$$\mu_{x,batch} = \frac{1}{SRT}\left(1 - \frac{p_{x,in,t}VS_{in,t}}{p_{x,out,t+\Delta t}VS_{out,t+\Delta t}}\right) \text{ (S5)}$$

Here, we can obtain a net specific growth rate that is no larger than the reciprocal of the SRT. SRT becomes an important boundary in shaping the microbial specific growth rate. The net growth rate was calculated for duplicate batches and averaged for representation.
